# Xenopatients show the need for precision medicine approach to chemotherapy in ovarian cancer

**DOI:** 10.18632/oncotarget.8325

**Published:** 2016-03-24

**Authors:** Jessica Erriquez, Martina Olivero, Gloria Mittica, Maria Stella Scalzo, Marco Vaira, Michele De Simone, Riccardo Ponzone, Dionyssios Katsaros, Massimo Aglietta, Raffaele Calogero, Maria Flavia Di Renzo, Giorgio Valabrega

**Affiliations:** ^1^ Department of Oncology, University of Torino, Candiolo, Torino, Italy; ^2^ Candiolo Cancer Institute, FPO-IRCCS Candiolo, Torino, Italy; ^3^ Department of Medical Sciences, University of Torino, Torino, Italy; ^4^ Department of Surgical Sciences, Gynecologic Oncology, AO-Universitaria Città della Salute, Torino, Italy; ^5^ Department of Molecular Biotechnology and Health Sciences, Molecular Biotechnology Center, University of Torino, Torino, Italy

**Keywords:** ovarian cancer, chemotherapy, patient derived xenograft

## Abstract

Platinum-based chemotherapy is the recommended first-line treatment for high-grade serous (HGS) epithelial ovarian cancer (EOC). However, most patients relapse because of platinum refractory/resistant disease. We aimed at assessing whether other drugs, commonly used to treat relapsed HGS-EOC and poorly active in this clinical setting, might be more effective against chemotherapy-naïve cancers. We collected couples of HGS-EOC samples from the same patients before and after neo-adjuvant platinum-based chemotherapy. Samples were propagated as Patient Derived Xenografts (PDXs) in immunocompromised mice (“xenopatients”). Xenopatients were treated in parallel with carboplatin, gemcitabine, pegylated liposomal doxorubicin (PLD) and trabectedin. PDXs derived from a naïve HSG-EOC showed responsiveness to carboplatin, trabectedin and gemcitabine. The PDXs propagated from a tumor mass of the same patient, grown after carboplatin therapy, did no longer respond to trabectedin and gemcitabine and showed heterogeneous response to carboplatin. In line, the patient experienced clinically platinum-sensitivity first and then discordant responses of different tumor sites to platinum re-challenge. Loss of PDX responsiveness to drugs was associated with 4-fold increase of NR2F2 gene expression. PDXs from another naïve tumor showed complete response to PLD, which was lost in the PDXs derived from a mass grown in the same patient after platinum-based chemotherapy. This patient showed platinum refractoriness and responded poorly to PLD as second-line treatment. PDX response to PLD was associated with high expression of TOP2A protein. PDXs demonstrated that chemotherapy-naïve HGS-EOC might display susceptibility to agents not used commonly as first line treatment. Data suggest the importance of personalizing also chemotherapy.

## INTRODUCTION

The majority of ovarian cancer patients are diagnosed with advanced disease for which cytoreductive surgery combined with platinum-based chemotherapy is the standard treatment [1]. Although the 5-year survival for women with advanced cancer has shown improvement, the majority of patients still die of the disease. Refractoriness or resistance to platinum-based chemotherapy is the main clinical issue. Currently, relapsed platinum-sensitive HGS-EOCs are successfully platinum re-challenged, while the optimal management of refractory/resistant cases is controversial [2]. Targeted agents have been approved for relapsed HGS-EOC such as Bevacizumab and Olaparib for BRCA1- and 2-mutated cancers. While these agents improved Progression Free Survival (PFS) in comparison with chemotherapy alone, no Overall Survival (OS) benefit was shown [3, 4]. On the other hand, large-scale genomic analyses have shown that HGS-EOCs lack recurrent actionable mutations [5, 6]. Therefore, it is of great interest to explore further the potential of so-called “traditional chemotherapies”.

A range of chemotherapeutic agents other than platinum drugs and taxanes has been approved for the treatment of ovarian cancer, such as doxorubicin, etoposide, topotecan, gemcitabine and trabectedin. These are mostly used as second-line drugs, but they resulted poorly effective in trials involving large unselected populations of relapsed patients. Thus, their choice for second-line treatment is usually based on drug's toxicity and patient's co-morbidity. Moreover, some of these drugs have been tested in trials of alternative first or second-line treatment, in combination with carboplatin or as third drug with carboplatin/paclitaxel, in comparison to carboplatin and paclitaxel. These alternatives did not result consistently superior [see e.g. refs. 7-10].

We and others [11-16] have undertaken the development of Patient-Derived Xenografts (PDXs) of ovarian cancers, based on the transfer of primary tumors directly from the patient into immunocompromised mice. PDXs can be propagated in large cohorts of tumor-bearing animals (“xenopatients“), which might receive not only the treatment devised for patients (co-clinical trial), but also simultaneously other drugs. The largest collections of ovarian cancers PDXs demonstrated that PDXs reflect patient experience [13, 15].

Here, we have used couples of samples derived from ovarian cancer patients before and after standard chemotherapy to test the efficacy of drugs approved for second-line therapy as treatment of naïve cancers.

## RESULTS

### Establishment in mice (xenopatients) of patient derived xenografts (PDXs) of ovarian cancer

**Figure 1:**
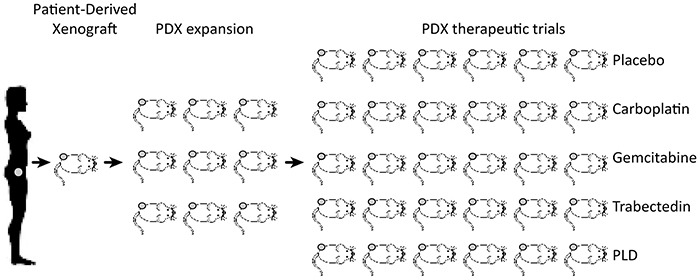
Schematic diagram of the engraftment and propagation of Patient Derived Xenografts (PDXs) and preclinical testing of chemotherapeutic drugs Sample from a patient is collected at either endoscopy, paracentesis or surgery and implanted subcutaneously in 1-3 mice. Successfully engrafted xenografts are harvested and re-implanted in a number of mice and eventually propagated to obtain cohorts of at minimum six mice for treatment.

PDXs have been established by sampling tumors at diagnosis and at cytoreductive surgery performed after either 3 or 6 cycles of neo-adjuvant chemotherapy. Thus, while patients have been treated according to standard international guidelines, cohorts of xenopatients became available to receive treatment (Figure 1). For this study, cohorts of xenopatients received drugs approved for the first- and second-line treatment of EOC.

Two cases of HGS-EOCs are reported here. Both patients were administered platinum-based neoadjuvant chemotherapy as first-line treatment. Patient 1 was treated with 6 cycles of carboplatin. She showed partial response, which persisted for more than 6 months, and then was platinum re-challenged at relapse. Patient 2 came out to be refractory to 3+3 cycles of neoadjuvant chemotherapy with carboplatin and taxol and was treated with pegylated liposomal doxorubicin (PLD) at relapse; she responded partially and transitorily to PLD as second-line treatment.

**Figure 2:**
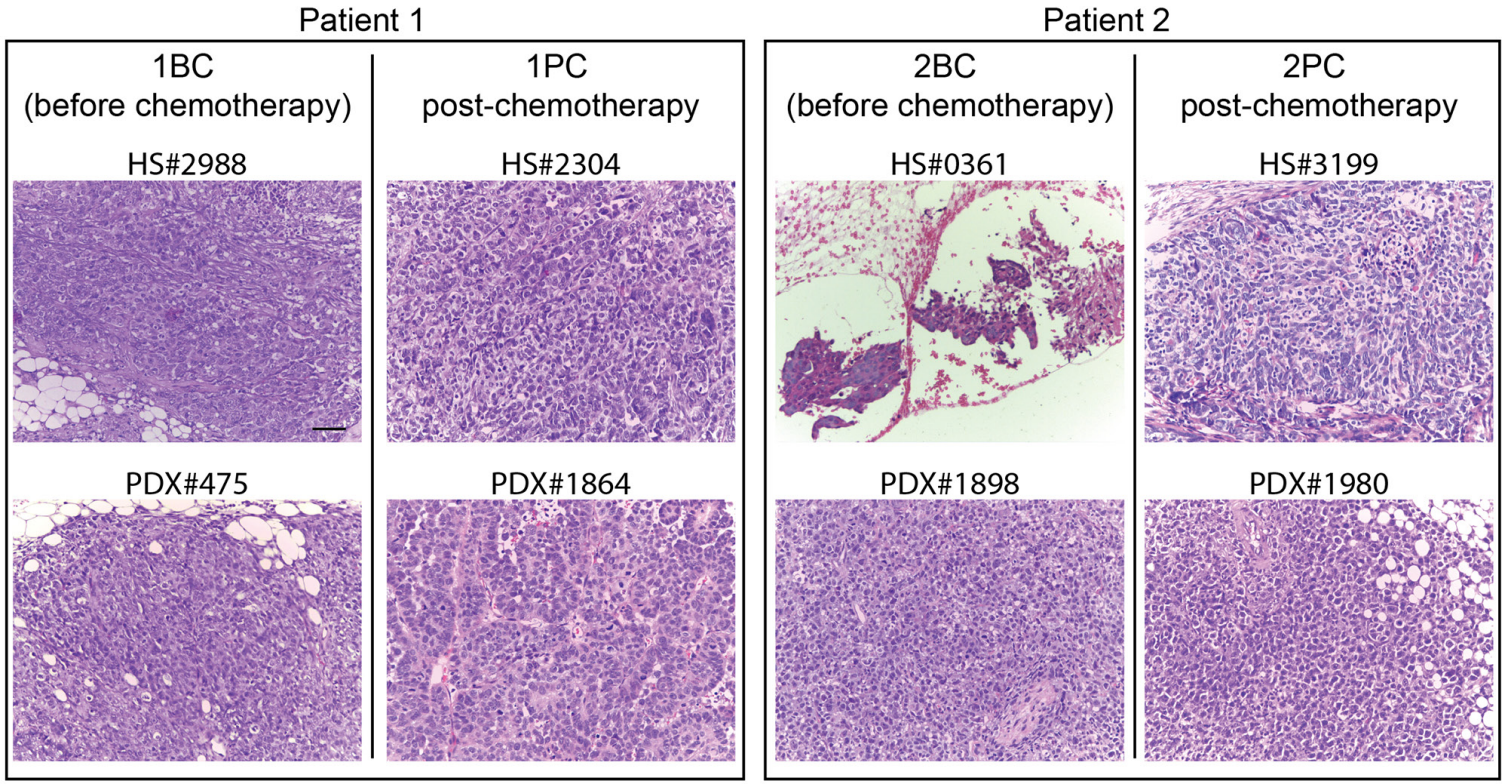
Histology of the originating tumors and the corresponding PDXs in xenopatients HS# indicates the serial number of the human sample from which the PDXs derive, as reported in the Profiling Protocol list. Bars: 50 μm. Below each human originating sample there is the corresponding PDX, indicated again by the serial number in the protocol. For each Patient: on the left, the originating sample harvested before the treatment of the patient with neoadjuvant chemotherapy and the relevant PDX; on the right the sample obtained after chemotherapy and the relevant PDX.

Two couples of PDX lines were derived from these patients, one obtained before chemotherapy and the second from mass grown after fist-line chemoterhapy. These were: from Patient 1 the PDX lines before (#1BC) and post-chemotherapy (#1PC), and from Patient 2 the PDX lines before (#2BC) and post-chemotherapy (#2PC). These PDX lines were propagated to obtain at least 40 mice/each (see e.g. Figure 1), to be randomized for the subsequent treatment. Original samples and the relevant PDXs were classified by the Pathologist as HGS-EOCs (Figure 2).

### Response of paired PDX lines to first-line and second-line chemotherapeutics

Xenopatients carrying each PDX line were randomized using the “Analysis Management Module” of the Laboratory Assistant Suite [LAS, 17], to obtain five comparable cohorts of 5-6 mice.

**Figure 3:**
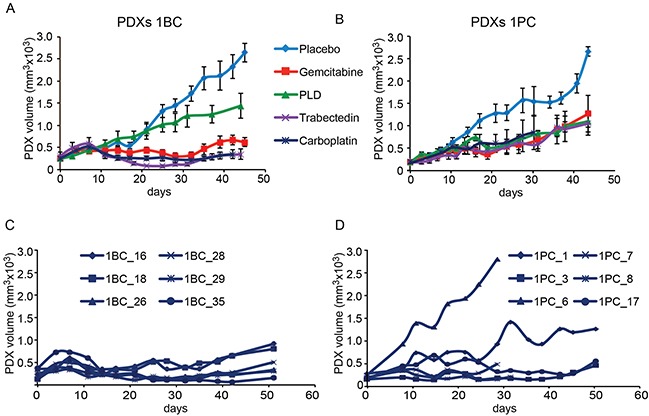
Treatment of the PDXs derived from Patient 1 with chemotherapeutic drugs The PDX line #1BC **A, C.** was obtained from the Patient 1′s sample before her treatment with platinum-based chemotherapy. The PDX line #1PC **B, D.** was obtained from a sample of the same Patient 1 at surgery performed after 6 cycles of platinum-based chemotherapy, to which the patient responded with a reduction of tumor burden. Panels A and B show the treatment of each cohort of 6 xenopatients as indicated (see also Figure 1). Panels C and D show the response to carboplatin of each PDX (numbered according to randomizing software) of the PDX cohorts obtained before (#1BC) and after (#1PC) platinum-based chemotherapy. Day 0 represent the beginning of mice treatment. Treatment details are reported in the Materials and Methods section.

**Figure 4:**
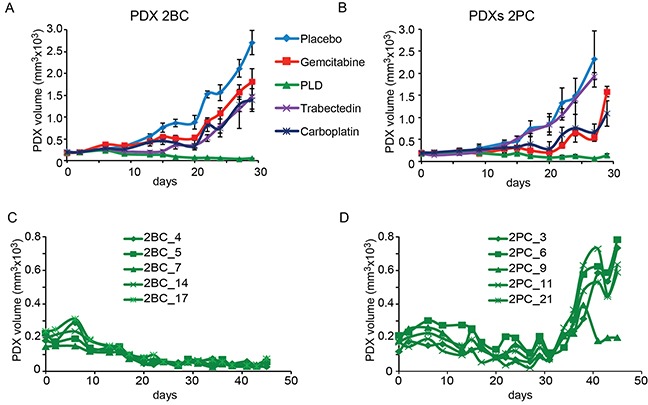
Treatment of the PDXs derived from Patient 2 with chemotherapeutic drugs The PDX line #2BC **A, C.** was obtained from the Patient 2′s sample before her treatment with platinum-based chemotherapy. The PDX line #2PC **B, D.** was obtained from a sample of the same Patient 2 at surgery performed after 3 cycles of platinum-based chemotherapy. Panels A and B show the treatment of each cohort of 6 mice as indicated. Panels C and D show the response to the prolonged treatment with PLD of the PDX cohorts derived from the two samples, before (#2BC) and after (#2PC) chemotherapy. Day 0 represent the beginning of mice treatment. Treatment details are reported in the Materials and Methods section.

As shown in Figures 1, 3 and 4, of each PDX line five cohorts were treated with either the placebo or one of the following chemotherapeutics: carboplatin, used commonly as first line therapy, and three drugs approved for second line treatment, i.e. gemcitabine, trabectedin and pegylated doxorubicin (PLD). Drugs were used at doses calculated on the basis of the standardized treatments of patients (see Material and Methods section).

Notably, both source Patients received carboplatin in first line treatment and were either re-challenged with carboplatin (the platinum-sensitive Patient 1), or with a second line drug, namely PLD (the platinum refractory Patient 2), as described in the Methods section.

As shown in Figure 3A the PDX line #1BC obtained from the naïve sample of Patient 1 responded not only to carboplatin but also to trabectedin and gemcitabine. The PDX line #1PC propagated from a sample harvested from the same Patient after platinum-based 6-cycle chemotherapy no longer responded to trabectedin and gemcitabine (Figure 3B). As far as carboplatin, we noticed that while the six mice bearing the PDXs #1BC responded consistently to carboplatin (Figure 3C), the response of the #1PC PDXs reflected high level of intra-tumor heterogeneity, being two PDXs out of six either not at all or poorly responsive (Figure 3D). This result explained the high variability of the median response to carboplatin of the post-chemotherapy PDX #1PC line (Figure 3B). Going back to the description of the clinical response of the Patient 1 to carboplatin re-challenge, it came out that some lesions, namely the lymph-node metastases, showed significant reduction, while other masses were not reduced by carboplatin and caused disease progression.

Figure 4 shows the response of the PDXs propagated from the paired samples of Patient 2. In this case, the PDXs derived from the naïve sample (#2BC) did not respond to all drugs but PLD (Figure 4A) and underwent durable shrinkage in all mice (Figure 4C). In line, the tumor of the Patient 2, treated with carboplatin and taxol was rated as platinum refractory. The response to drugs of the #2PC cohorts, obtained from a tumor mass grown after first-line platinum based-chemotherapy is shown in Figure 4B. The short-term response to all drugs looked superimposable (Figure 4B) to that of the PDXs derived from the naïve tumor. However, the response to PLD was partial, as the #2PC PDXs underwent volume stabilization in the first 30 days, but re-grew in the following days (Figure 4D).

**Figure 5:**
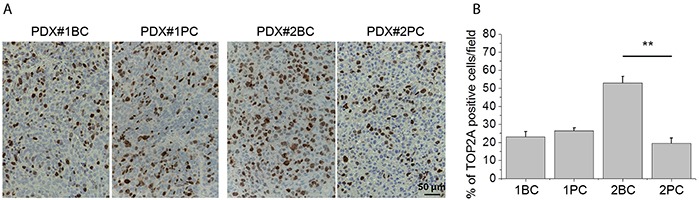
Expression of TOP2A protein in the PDXs **A.** Representative images showing the immunohistochemical detection of TOP2A protein in the indicated PDX line. Bars: 50 μm. **B.** Percentage of cells showing detectable staining with TOP2A antibody, measured using the NIH ImageJ software. The TOP2A signal was estimated by the ratio between the area occupied by positive cells and the total area occupied by cancer cells in the same field. Statistical significance was determined using ANOVA test. **P < 0.01.

The susceptibility of the PDX line #2BC to PLD was associated with the over-expression of the TOP2A protein (Figure 5), as it has been demonstrated also previously [11]. The PDX line derived from the same Patient's sample after chemotherapy (#2PC], which had lost susceptibility to PLD was made of cells expressing very low level of nuclear TOP2A, comparable to that of the PDXs showing resistance to PLD from the beginning, such as the PDX lines #1BC and #1PC (Figure 5).

### Expression profiling of PDXs obtained before and after patient treatment with platinum-based chemotherapy

The molecular make-up of PDXs generated from tumor samples obtained before and after platinum-based chemotherapy has been analyzed using expression profiling.

**Figure 6:**
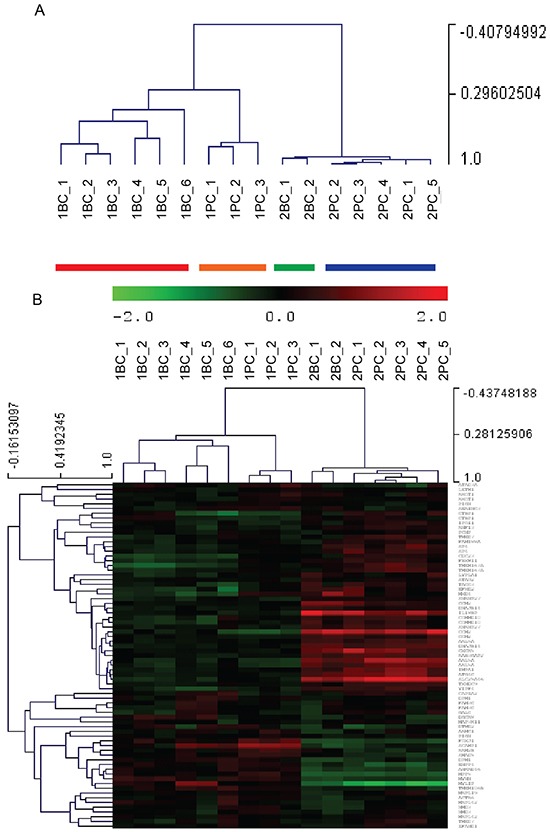
Global expression profiling of PDXs propagated from the two Patients’ samples harvested before and after chemotherapy **A.** Unsupervised hierarchical clustering shown as dendrogram (clustering tree) derived from expression levels of 11,866 gene probes in the individual PDXs obtained from Patient 1′s (#1BC and #1PC) and Patient 2′s (#2BC and #2PC) samples. Individual PDXs were numbered by the randomizing software. The vertical axis on the right shows the level of similarities between PDXs. **B.** Heat map showing the unsupervised hierarchical clustering of the PDXs by means of 73 genes, selected among the 129 genes which defined the profiles of platinum-sensitive versus the platinum resistant HGS-EOCs analyzed by Patch et al. [6].

For each PDX line 2-6 xenografts of the P1-P3 passages were sampled for mRNA extraction, cRNA generation and hybridization to microarray. The dendrogram (Figure 6A) generated using the unsupervised clustering of the 11,866 probes showed that expression profiles distinguished the PDX lines generated from the two patients, who differed as far as platinum sensitivity. Moreover, the dendrogram shows also the separation between PDXs derived from the same patient's samples before and after chemotherapy (Figure 6A).

The separation of platinum-sensitive and platinum resistant PDXs was confirmed using a dataset recently published by Patch et al. [6]. The latter Authors have reported the whole-genome DNA and RNA sequencing data of HGS-EOC from 92 patients, who received platinum-based chemotherapy as part of primary treatment, and were classified as either refractory or resistant or sensitive to primary treatment. These Authors have defined an RNAseq transcription signature of sensitive versus resistant tumors made of 129 genes. We have used 73/129 genes, whose probes are present in our dataset, to cluster all our PDX samples (Figure 6B). These 73 genes allowed the separation of the platinum-sensitive versus resistant PDXs from the two patients and also of the subset of PDXs obtained from the same patients before and after chemotherapy. Moreover, 19 out of the 30 top ranked set of genes that mainly described the differences between the two patients in our dataset were characterized by expression variation, which replicated that of the 129 differentially expressed genes in the published data [6].

Among the top differentially expressed genes that separated our platinum-sensitive PDX lines (#1BC and #1PC) from the resistant ones (#2BC and#2PC), we identified IRF-1 (Interferon Regulatory Factor 1) as the most reliable marker of platinum sensitivity. Interestingly, we have identified IRF-1 previously as a gene associated with the response of ovarian cancer cells to platinum [18]. Moreover, IRF-1 was also present in the list of 94 genes, which separated sensitive from refractory/resistant HGS-EOCs in the above-mentioned whole genome-wide expression analysis [6].

The comparison of the platinum refractory PDXs lines obtained from Patient 2 before (#2BC) and after chemotherapy (#2PC) demonstrated that the two lines were only modestly distinguishable in global expression profiling. This is shown not only by the dendrogram of Figure 6A but also by the short list of the differentially expressed genes (15 genes, see Supplementary Figure S1).

Conversely, the comparison between the #1BC and #1PC PDX lines from Patient 1 showed the differential expression of 54 genes (Supplementary Figure S1), being the NR2F2 gene the most up-regulated (>4-fold). The 54 genes were also submitted to the Ingenuity Knowledge Base to link genes to known functional pathways, and to identify gene-to-gene interaction networks. Forty-nine genes out of 54 were mapped with the Ingenuity Pathway knowledge base. The summary of the over-represented Ingenuity Pathway Analysis (IPA) Diseases and Biological Functions is reported in the Supplementary Figure S2. Data showed that not only proliferation related genes but also motility and migration related genes were over-represented and linked to drug resistance. This came out to be particularly interesting as it has been recently demonstrated that cell motility/migration might be the key to tumor growth and drug resistance [19].

## DISCUSSION

Data reported here show that PDXs allowed to disclose the susceptibility of chemo-naïve ovarian cancer to drugs, namely trabectedin, gemcitabine and pegylated doxorubicin that are definitely less commonly used as first line therapy than in successive lines of treatment. Most importantly, the samples harvested and propagated as PDXs from the same patient after standard platinum-based chemotherapy, were no longer susceptible to the above drugs.

The use of PLD in primary and relapsed EOC has been tested in Phase III trials [7, 8, 20]. Its addition in first-line or second-line treatment did not improve overall survival (OS). Similarly, OS was not improved by the addition of gemcitabine to carboplatin and paclitaxel in first line treatment [10]. Trabectedin was never tested in naïve ovarian cancer patients but only in second- or third-line of treatment. All the above studies have been carried out in large unselected patients’ populations while data shown here suggest that more accurate selection is worth being explored to identify possible alternatives as first-line treatment.

The expression profiling of the coupled PDX lines from the two cases shows once more the high level of inter-tumor heterogeneity of HGS-EOC, which current histological classification does not show and has been comprehensively demonstrated by whole genome studies [5, 6].

Conversely, the signatures of platinum resistant EOCs identified by the most recent, comprehensive, genome-wide expression analysis of HGS-EOC [6] were able to separate even the small number of PDX samples of our study. Among the genes included in one of these signatures, IRF-1 was found as the most differentially expressed in our platinum-sensitive versus platinum resistant PDXs. The Interferon Regulatory Factors (IRFs) are transcription factors involved in immune responses and oncogenesis and most of them are classified as tumor suppressors. The expression and activation of IRF(s) are stimulated by several cytokines and by DNA damage. We have already shown also that IRF-1 expression is functionally correlated to platinum resistance of ovarian cancer cells as it is 2-fold increased after cisplatin treatment and limits cell response to this drug [18].

The heterogeneous response of individual PDXs of one cohort to carboplatin reproduces the intra-tumor heterogeneity (ITH) of ovarian cancer, which has been shown already by genetic studies [see e.g. refs. 21-23]. The ITH of this PDX line reflected the heterogeneous response of the relapsed tumor of the same patient to re-challenge with carboplatin.

While the concept of patients’ selection and personalized treatment is universally accepted when targeted therapies are trialed, the possibility of selecting patients for chemotherapy is largely neglected. It is noteworthy that in most instances biomarkers predict resistance rather than susceptibility to chemotherapeutic drugs. In EOC too, a range of molecular changes have been associated with acquired chemoresistance rather to responsiveness [6]. Biomarkers of response to chemotherapeutics are more difficult to identify, also because most of the drugs inhibit DNA replication. However, some of the conventional chemotherapeutics have known molecular targets. These are for example anthracyclines that inhibit topoisomerases. In line, we have recently shown that topoisomerase II gene copy gain predicts response of platinum refractory/resistant EOC cancers to PLD [11], as well as being associated with the response to PLD in breast cancer [24, 25]. We show here that the PDXs propagated from a naïve tumor showed responsiveness to PLD that was lost in the post-chemotherapy sample of the same patient. As the former displayed TOP2A high expression and the latter did no longer express similar levels of TOP2A, our data corroborate the suitability of TOP2A as biomarker of response to PLD, which we previously proposed [11].

NR2F2 was found overexpressed in the PDX lines from a post-chemotherapy sample, which was first susceptible and then became resistant to gemcitabine and trabectedin. This gene has been already linked to ovarian cancer onset and progression, as it was found amplified in 7% of the 311 HGS-EOC reported in TCGA [5] and in 20% of 80 primary HGS-EOC studied by Patch and co-workers [6]. This gene has been previously found up-regulated in platinum resistant ovarian cancer cells [26] and has been strongly associated to poor prognosis pancreatic cancer [27], which is usually treated with gemcitabine.

Our data confirm the value of PDXs as preclinical models and allowed to reveal the potential and unexpected susceptibility of naïve cancers to PLD, gemcitabine and trabectedin. Conversely, there are substantial limitations to the use of the PDX model for current clinical decision making [summarized in 28], although pilot studies have reported clinical good responses to PDX-guided choice of chemotherapeutics [29, 30]. However, the clinical impact of these models on precision medicine may be substantial through the elucidation of biomarkers of sensitivity or resistance with the aim of identifying strategies for future patients.

In summary, oncologists have achieved little progress in tailoring chemotherapy for cancer. In ovarian cancer, cytoreductive surgery associated with platinum-based chemotherapy remains the standard of care for both patients with limited and advanced disease. However, since in the long term about 70% of EOC manifests refractoriness/resistance to platinum, data showing that each tumor might display selective susceptibility to other chemotherapeutic drugs indicate that also conventional chemotherapy could be more effectively tailored.

## MATERIALS AND METHODS

### Patients’ characteristics

We have collected paired tumor samples (naïve and post-chemotherapy) from EOC patients treated at the Candiolo Cancer Institute FPO-IRCCS and in the S. Anna Hospital of the Città della Scienza e della Salute di Torino. Samples were implanted, propagated and characterized according to the “Profiling” Protocol approved by the local Ethical Committee. The in depth study of two cases is reported here.

Patient 1 of this study was 75 years old and underwent ileostomy following bowel obstruction for suspected tumor of the left ovary. During surgical operation, peritoneal washing and multiple omental and peritoneal biopsies were performed; at definitive histology HGS-EOC was diagnosed. From an omental node we obtained the PDX line #1BC (Before Chemotherapy), corresponding to the PDX line #475 of the Protocol. Since the patient was not eligible for debulking surgery due to the presence of diffused peritoneal carcinomatosis (clinical stage FIGO IIIC) and she had significant comorbidity, she was treated with neoadjuvant chemotherapy regimen consisting of carboplatin alone. Following three cycles of chemotherapy, the computed tomography (CT) scan compared with baseline CT, revealed shrinkage of the ovarian lesion and stability of peritoneal carcinomatosis. Moreover, reduction of the serum marker CA125 was observed. Additional three cycles were administered. We observed disease stability and further reduction of CA125, which was 109 U/ml before chemotherapy, 10 U/ml after three cycles and 4 U/ml at the end of the treatment. After these 6 platinum-based cycles, the patient underwent non optimal cytoreductive surgery (residual tumor or R>1 cm). The PDX line #1PC (Post Chemotherapy), corresponding to the PDX line #1864 of the protocol, was obtained from the left ovary removed during this surgery. After 6 months, disease progression (in the liver, abdominal lymph nodes and pelvis) was observed at CT scan; a subsequent second line platinum-based chemotherapy was performed with liver and pelvic progression and shrinkage of pelvic and lomboaortic lymph nodes after 6 cycles. The patient died 4 months after the conclusion of chemotherapy.

From the ascites sample of the naïve Patient 2 we obtained the PDX line #2BC (Before Chemotherapy), corresponding to the PDX line #1898 of the Protocol. This patient was a 62 years old woman, who presented at the Emergency Room with abdominal pain and dyspeptic symptoms; the abdomen CT scan showed the presence of a pelvic mass along with diffused omental and peritoneal carcinomatosis. At laparoscopy an omental biopsy of the mass showed the presence of HGS-EOC (clinical stage FIGO IIIC). CA125 was 1,256 U/mL at diagnosis. As the tumor was not susceptible of primary optimal cytoreduction because of diffused peritoneal carcinomatosis, the patient was treated with neoadjuvant chemotherapy regimen consisting of carboplatin and paclitaxel. Following three cycles of chemotherapy, the CT scan revealed partial response of peritoneal carcinomatosis. CA125 decreased to 275 U/mL. Thus, the patient underwent Interval Debulkyng Surgery (IDS). Uterus, ovaries, multiple peritoneal biopsies, pelvic and lomboaortic lymph nodes were removed. However, cytoreductive surgery was suboptimal (R> 1 cm). From an omental node the #2PC (Post Chemotherapy) PDX line, corresponding to the PDX#1980 of the Protocol, was obtained. Three additional cycles with carboplatin and paclitaxel were performed. Three months after postoperative chemotherapy, CT scan showed peritoneal progression. CA125 was increased to 567 U/mL. Second line treatment with pegylated liposomal doxorubicin (PLD) was started and after 2 cycles significant reduction of CA125 was observed (217 U/mL); moreover, CT scan showed disease stability in the peritoneum with only modest increase of ascites. Chemotherapy with PLD was withdrawn for G2 skin and G3 gastrointestinal toxicity after only 3 cycles. Third line treatment with gemcitabine was endeavored, but after 2 cycles the patient displayed peritoneal progression and eventually died 5 months later.

### Patient derived xenografts

Samples (naïve and post-chemotherapy) were obtained from patients such as those described above and implanted subcutaneously in the right flank of severely immunocompromised NOD/Shi-scid/IL-2R γnull mice. In our series the take rate was 47%.

Samples from surgery, such as those that gave rise to the PDX lines #1BC, #1PC and #2PC, were examined and selected by the pathologist free of necrotic tissue. We implanted subcutaneously in mice tumor samples of 125 mm^3^, plunged in Matrigel® (BD Biosciences) containing medium. The ascites that gave rise to the PDX line #2BC was washed, pelleted and examined by the pathologist. An aliquot of pellet containing about 7*10^6^ tumor cells was injected subcutaneously as above. As shown in Figure 1, xenografts were propagated in two generations of mice and, for each PDX line, cohorts of six mice were randomized using the “Analysis Management Module” of the Laboratory Assistant Suite [LAS, 17], from approximately 40 mice per line with established tumors (average volume 200 mm^3^). Cohorts were treated with the following regimens: PLD (Lipodox, Sun Pharmaceutical Industries Cranbury, NJ 08512) 3 mg/kg, prepared in 5% glucose solution, administered once through the tail vein; carboplatin (Teva Pharmaceuticals, USA) 25 mg/kg once-weekly for 4 weeks administered via intraperitoneal injection; gemcitabine (Teva Pharmaceuticals, USA) 100 mg/kg twice-weekly for 4 weeks administered via intraperitoneal injection; trabectedin (Yondelis®, Pharma Mar, Spain) 200 μg/kg once administered through the tail vein.

Tumor size was evaluated twice-weekly with digital caliper and volume was calculated using the formula 4/3π*(d/2)^2^*D/2, where d is the minor tumor axis and D is the major tumor axis. All animal procedures were approved by the Ethics Committee of the Candiolo Cancer Institute and by the Italian Ministry of Health.

The following samples have been stored from PDXs: frozen samples (in Fetal Bovine Serum, plus 10% DMSO, Sigma Aldrich, USA) in liquid nitrogen for subsequent propagation, RNAlater® (Thermo Fisher Scientific, USA) embedded samples for molecular analyses and formalin-fixed, paraffin embedded (FFPE) samples for morphological analyses.

### Gene expression analysis

Total cellular RNA was isolated from samples stored in RNAlater, using the SV Total RNA Isolation kit (Promega, Fitchburg, WI, USA). The RNAs were then quantified and inspected by bioanalyzer analysis (Agilent Technologies, Waldbrom, Germany). Complementary RNAs were generated and hybridized according to the Illumina Total Prep RNA Amplification Protocol (Illumina, San Diego, CA, USA). Data were obtained using the HumanHT-12 version 4 Illumina bead array technology. The transcript average intensities were calculated using Illumina BeadStudio software, and were normalized by the Rank-Invariant Method. Normalized data were log_2_ transformed and centered to the median. Principal component analysis and hierarchical clustering (ST, Euclidean distance, average clustering, 5000 jackknife resampling steps) were performed using the Multi Experiment Viewer 4.9.0 application [31]. Differentially expressed genes were identified using the SAM methods implemented in the above program. Ingenuity Pathway Analysis (www.ingenuity.com) was performed for the interpretation of the biological significance of the observed differentially expressed genes.

### Immunohistochemistry

Immunohistochemical studies were performed as described [32] to detect topoisomerase II alpha (TOP2A) using monoclonal rabbit antibody (D10G9, Cell Signalling Technology), in paraffin-embedded tumor material. Ten images of each sample were then acquired by optical microscope (20x) connected with CCD camera. The images were analyzed using the NIH ImageJ (W. Rasband, NIH) software. The TOP2A signal was estimated by the ratio between the area occupied by positive cells and the total area occupied by cancer cells in the same field [33].

### Statistical analysis

Statistical analysis of the data was performed using ANOVA (Microsoft Excel; Microsoft, Redmond, WA, USA).

## SUPPLEMENTARY FIGURES


