# Supplementary material for: Xenopatients show the need for precision medicine approach to chemotherapy in ovarian cancer
Source: Oncotarget. 2016 Mar 24;7(18):26181–91. doi: 10.18632/oncotarget.8325 (PMC5041973; doi:10.18632/oncotarget.8325)
Supplement: Supplementary file 1 [file oncotarget-07-26181-s001.pdf]

SUPPLEMENTARY FIGURES

A

Significant Genes	more than 2 fold change
475-1864/1898-1980	1696
1864/475	54
1980/1898	15

B

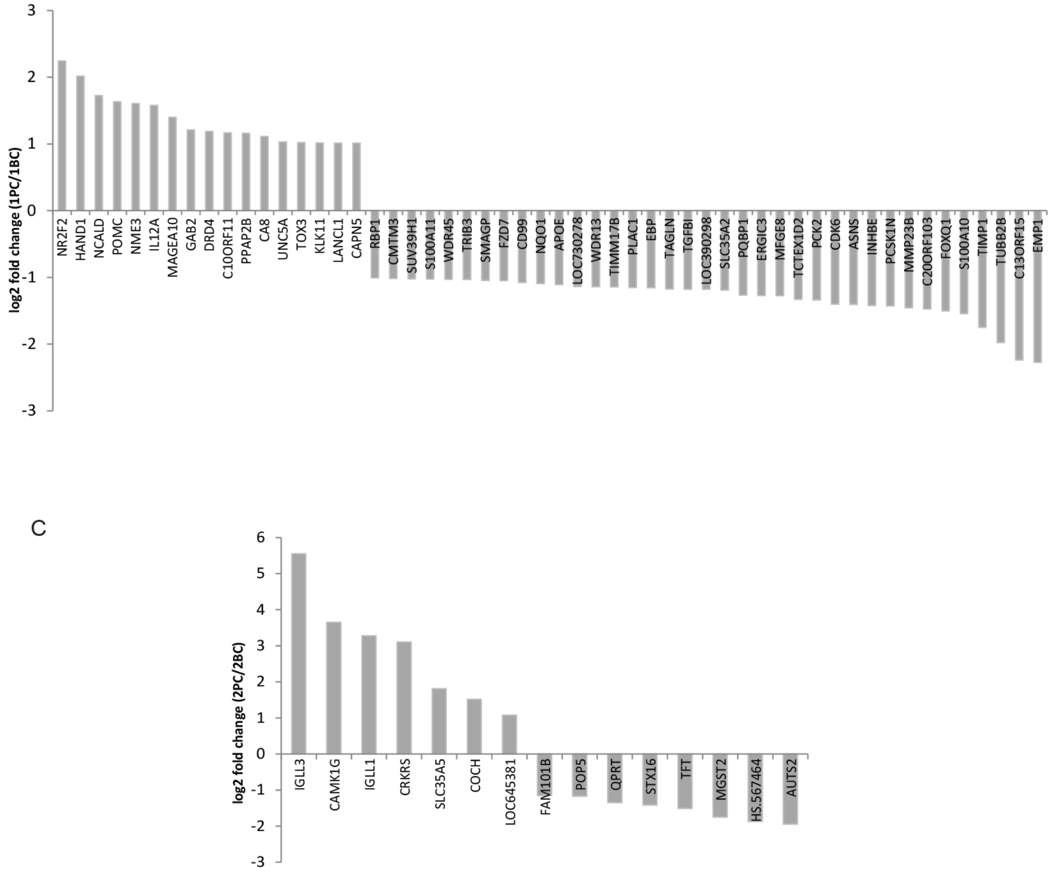

Supplementary Figure S1: Differentially expressed genes from the comparison of the PDX lines. A. Number of genes differentially expressed more than 2 fold as from the indicated comparisons; **B.** bar chart of the 54 genes differentially expressed more than 2 fold in the #1PC PDXs versus the #1BC PDXs; **C.** bar chart of the 15 differentially expressed more than 2 fold in the #2PC PDXs versus #2BC PDXs.

Diseases and Bio Functions

Categories	Functions	Diseases or Functions Annotation	p-Value	Molecules	# Molecules
Cellular Movement	cell movement	cell movement	2.37E-07	S100A11, CD99, APOE, GAB2, IL12A, TGFBI, UNC5A, NR2F2, NQO1, CDK6, HAND1, POMC, ASNS, RBP1, TUBB2B, PLAC1, TIMP1, PPAP2B, FOXQ1, S100A10, FZD7	21
Cellular Growth and Proliferation	proliferation	proliferation of cells	9.57E-07	S100A11, GAB2, APOE, CD99, IL12A, UNC5A, SUV39H1, NR2F2, HAND1, MFGE8, POMC, ASNS, RBP1, PCK2, PLAC1, TIMP1, S100A10, TGFBI, NQO1, CDK6, TRIB3, PCSK1N, EMP1, NME3, TAGLN, FOXQ1, FZD7	27
Cellular Movement	migration	migration of cells	4.79E-06	S100A11, CD99, GAB2, APOE, IL12A, TGFBI, NR2F2, NQO1, CDK6, POMC, ASNS, TUBB2B, PLAC1, TIMP1, PPAP2B, FOXQ1, S100A10, FZD7	18
Cancer, Organismal Injury and Abnormalities	advanced malignant tumor	advanced malignant tumor	2.45E-05	EMP1, S100A11, CD99, APOE, KLK11, IL12A, TIMP1, PPAP2B, NR2F2, CDK6, FZD7	11
Metabolic Disease	amyloidosis	amyloidosis	2.62E-05	APOE, GAB2, EBP, TGFBI, TIMP1, NQO1, TAGLN, MFGE8, DRD4	9
Cancer, Organismal Injury and Abnormalities, Tumor Morphology	progression	progression of malignant tumor	2.70E-05	APOE, TIMP1, NR2F2, CDK6	4
Cellular Development, Cellular Growth and Proliferation	proliferation	proliferation of tumor cell lines	2.84E-05	CD99, GAB2, IL12A, SUV39H1, NQO1, CDK6, HAND1, POMC, TRIB3, ASNS, EMP1, PLAC1, PCK2, FOXQ1, S100A10	15
Metabolic Disease	metabolism	abnormal metabolism	3.75E-05	APOE, NQO1, FOXQ1, POMC, S100A10	5
Cellular Movement, Immune Cell Trafficking	invasion	invasion of leukocytes	3.85E-05	TIMP1, NQO1, S100A10	3
Cardiovascular System Development and Function	neovascularization	neovascularization	4.53E-05	APOE, IL12A, TIMP1, MFGE8, S100A10	5
Cancer, Organismal Injury and Abnormalities	metastasis	metastasis	5.22E-05	EMP1, S100A11, CD99, APOE, IL12A, TIMP1, PPAP2B, NR2F2, CDK6, FZD7	10
Lipid Metabolism, Small Molecule Biochemistry	regulation	regulation of lipid	7.28E-05	APOE, POMC, RBP1	3
Organismal Injury and Abnormalities, Tissue Morphology	quantity	quantity of lesion	8.02E-05	APOE, IL12A, TIMP1, NQO1, MFGE8	5

Supplementary Figure S2: Ingenuity Pathway Analysis (IPA) of the genes differentially expressed more than 2 fold in the #1PC PDXs versus #1BC PDXs. Over-represented IPA Diseases and Biological Functions associated to 49 out of 54 genes differentially expressed.
